# Effect of *Elettaria cardamomum* L. on hormonal changes and spermatogenesis in the propylthiouracil‐induced hypothyroidism male BALB/c mice

**DOI:** 10.1002/edm2.438

**Published:** 2023-07-04

**Authors:** Bahar Atabaki, Naser Mirazi, Abdolkarim Hosseini, Abdolrahman Sarihi, Zahra Izadi, Alireza Nourian

**Affiliations:** ^1^ Department of Biology, Faculty of Basic Sciences Islamic Azad University Hamedan Iran; ^2^ Department of Biology, Faculty of Basic Sciences Bu‐Ali Sina University Hamedan Iran; ^3^ Department of Animal Sciences and Biotechnology, Faculty of Life Sciences and Biotechnology Shahid Beheshti University Tehran Iran; ^4^ Department of Neuroscience, School of Sciences and Advanced Technology in Medicine Hamadan University of Medical Sciences Hamadan Iran; ^5^ Department of Horticulture Sciences and Engineering, Nahavand Higher Education Complex Bu‐Ali Sina University Hamedan Iran; ^6^ Department of Pathobiology, Faculty of Veterinary Science Bu‐Ali Sina University Hamedan Iran

**Keywords:** *Elettaria cardamomum*, hypothyroidism, spermatogenesis

## Abstract

**Introduction:**

Spermatogenesis is significantly influenced by the thyroid gland. Thyroid disorders can be caused by a variety of factors. Traditionally, *Ellettaria cardamomum* has been used to treat a variety of ailments. The effects of *E. cardamomum* extract (ECE) on spermatogenesis in hypothyroid mice were investigated in this study.

**Methods:**

In this study 42 male mice, weighing (25–35 g) were randomly divided in six groups: control group (taking normal saline, 0.5 mL/day, by oral gavage [P.O.]), hypothyroid group (taking 0.1% propylthiouracil in drinking water for 2 weeks), hypothyroid groups treated by levothyroxine (15 mg/kg/day, P.O.) and hypothyroid groups treated by ECE (100, 200 and 400 mg/kg/day, P.O.). After the end of experiments the mice were anaesthetised and blood samples were collected for hormonal analysis.

**Results:**

The sperm count and microscopic studies of testes were done also. Our results showed that the T_3_, T_4_, testosterone levels and spermatogenesis in hypothyroid animals decreased and thyroid‐stimulating hormone, follicle‐stimulating hormone and luteinizing hormone increased compared with control group. Treatment by ECE reverse these effects in comparison with hypothyroid group.

**Conclusions:**

According to our findings, the ECE may stimulates thyroid gland function and increases testosterone and spermatogenesis.

## Graphical abstract

Spermatogenesis is significantly influenced by the thyroid gland. Our results showed that the T_3_, T_4_, testosterone levels spermatogenesis in hypothyroid animals decreased and thyroid‐stimulating hormone, follicle‐stimulating hormone and luteinizing hormone increased compared with the control group. Treatment by *Ellettaria cardamomum* extract reverse these effects in comparison with hypothyroid group.

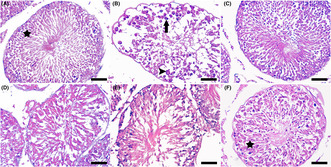

## 1 INTRODUCTION

Infertility and related problems are known as one of the important issues in the life of couples.^1^ According to the available statistics, 35% of couples' infertility cases are related to men and 25% of infertility cases are related to both couples. The most common cause of male infertility is their inability to produce a sufficient number of healthy and active sperms and disturbances in the spermatogenesis process.^2^, ^3^ The binding of follicle‐stimulating hormone (FSH) to Sertoli cells causes the synthesis of androgen‐binding protein (APB).^4^ Testosterone is secreted by Leydig cells. These cells are located in the interstitial tissue between the spermatogenic tubes. The amount of testosterone synthesis and secretion is regulated by the amount of luteinizing hormone (LH) in the blood. The level of testosterone secretion is directly related to the level of LH.^5^

Thyroid gland function disorders and related hormonal fluctuations can each affect the body's chemical interaction mechanism. As long as the disturbances resulting from these fluctuations lead to irreparable damage that endangers natural life. Thyroid hormones, which were previously thought to have no effect on spermatogenesis, are now recognized as an important factor influencing this process. The identification of thyroid hormone receptors on Sertoli cells in the testis initiated further studies to investigate the role of these hormones in male reproduction.^6^ The effects of changing thyroid hormones on reproductive systems have been widely studied in humans and animal models, and the results have generally shown that changing thyroid function from the normal state leads to a decrease in fertility.^7^

Medicinal plants are of special interest as a potential source of new chemotherapeutic drugs due to having a wide spectrum of bioactive compounds; because herbal medicines, due to their natural origin, are more compatible with living organisms, including the human body, than chemical medicines and cause fewer side effects.^8^ The green cardamom plant with the scientific name *Elettaria cardamomum* is an herbaceous, perennial and evergreen plant. The origin of this plant is reported in South India and Sri Lanka.^9^ *E. cardamomum* fruit is rich in volatile oils, which mainly include phenolic and flavonoid compounds. Starch, protein, waxes and sterols are other compounds found in green cardamom extract.^10^ Phenolic compounds extracted from cardamom fruit have been proven to have antibacterial activity against *Staphylococcus aureus* and *Escherichia coli* extracted from prostate glands.^11^ Kumari and Dutta^12^ stated that the use of cardamom with a dose of 2% effectively reduces physiological changes and improves parenchymal levels in the lungs. In another study, it was shown that the use of green cardamom extract improves lung congestion, asthma, heart disease, eyelid inflammation and abdominal insufficiency.^13^

Considering that the oral consumption of this plant has been common among people for a long time, and its therapeutic use in traditional medicine, and so far, there are no studies on the therapeutic effects of this plant. The effect of the plant on the process of spermatogenesis in patients with hypothyroidism has not been done, this study design to investigate the protective effect of *E. cardamomum* on spermatogenesis process in the hypothyroid male mice.

## 2 MATERIALS AND METHODS

### 2.1 Plant material and extraction

The *E. cardamomum* plant was purchased from Avicenna's Medicinal Plants Center of Hamedan Province Agricultural Jihad Organization (herbarium code: 36586) in the early summer of 2014. Since the prepared plant was fresh, it was spread on a paper for a few days and completely dried. Then the dried seeds were powdered with an electric mill. To prepare the extract 200 g of the seed powder was poured into 1000 mL of ethanol and place in the dark room for 1 week; then, the resulting mixture was filtered and concentrated in vacuum at 45°C using a rotary apparatus (EYELA). The resultant extract was dried and stored in the refrigerator at 4°C until the experiment. The ECE prepared as 100, 200 and 400 mg/kg of body weight of animal and were given by oral rout (P.O.). The selected dose based on earlier report.^14^

### 2.2 Preparation of a solution containing 0.1% propylthiouracil

In order to induce hypothyroidism in mice, the method of feeding propylthiouracil (PTU) in drinking water was used. To prepare the above solution, 0.1 mg of each tablet was dissolved in 100 mL of drinking water and given to the animals. Daily, the above solution was freshly prepared and freely provided to the mice. After 2 weeks, rats consuming this solution will develop hypothyroidism.^15^

### 2.3 Preparation of levothyroxine solution

Levothyroxine drug was obtained in the form of oral tablets containing 0.1 mg of the active ingredient (Iran Hormone Pharmaceutical Company). Then each tablet was dissolved in 20 mL of distilled water. The resulting solution will have a concentration of 5 μg/mL. From the resulting solution, each animal was fed with a dose of 15 μg/kg and with a certain volume of 0.2 mL by insulin syringe to each mouse daily and by gavage.^16^

### 2.4 Animals' maintenance and ethical considerations

In this experimental study, 42 male BALB/c mice weighing 25–35 g and six‐weeks‐old were purchased from Pasteur Institute in Tehran. The animals were kept in a suitable place in terms of humidity and temperature, and their cages were cleaned regularly. Animals had free access to food and water. All mice were kept in standard cages with a temperature of 22–25°C and suitable humidity for 1 week in the animal house of Bu Ali Sina University with 12 h of darkness and 12 h of light. All stages of this thesis and work with laboratory animals were carried out under the standard conditions and guidelines of the International Ethics Committee for Working with Laboratory Animals and the Ethics Committee of Islamic Azad University, Hamedan branch (permission number: IR.IAU.H.REC.1395.027).

### 2.5 Study design and grouping

The animals were randomly divided into six groups as follows:

- *Control group*: Treatment with physiological normal saline in the amount of 0.2 mL (P.O.) for 10 days.
- *PTU group*: Causing hypothyroidism in them by using the drug PTU at the rate of 0.1% in drinking water for 2 weeks and treatment with physiological normal saline in the amount of 0.2 mL (P.O.) for 10 days.
- *LEVO group*: Hypothyroid and treated with levothyroxine (15 μg/kg, daily, P.O.) for 10 days.
- *ECE 100 group*: Hypothyroid and treated with hydroalcoholic extract of *E. cardamomum* at the dose of 100 mg/kg P.O. for 10 days.
- *ECE 200 group*: Hypothyroid and treated with hydroalcoholic extract of *E. cardamomum* at the dose of 200 mg/kg P.O. for 10 days.
- *ECE 400 group*: Hypothyroid and treated with hydroalcoholic extract of green cardamom fruit at the dose of 400 mg/kg P.O. for 10 days.

### 2.6 Blood and tissue sampling

The animals were anaesthetised by ketamine (Alfasan Co., 80 mg/kg) and xylazine (Alfasan Co., 10 mg/kg). Blood sampling was done directly from the heart at 10:00 AM using a sterile 5 cc syringe and immediately after the end of the blood collection, the tubes were placed in the centrifuge. The device set to 10 min with 1700 *g* and afterward serum transfer into the special microtubes. Serum used to determine the concentration of T_3_, T_4_, thyroid‐stimulating hormone (TSH), testosterone, LH and FSH hormones.

In addition, the testicular tissue sample was separated from the animals and after weighing and examining the morphological characteristics, it was immediately placed in 10% formalin solution to fix and prepare histological sections. Also, epididymal tissue samples of the left testicle were separated from each animal and separated in physiological serum at 37°C for sperm count. Also, the gonadosomatic index (GSI) was expressed as the percentage of the total body weight in relation to the organ weight (GSI = [organ weight/total body weight] × 100%).

### 2.7 Hormone measurement methods

One of the hormone measurement methods is the enzyme‐linked immunosorbent assay (ELISA) method, which consists of a two‐step enzymatic reaction with the sandwich method, and at the end of the test, instead of a coloured product, a fluorescent product is created. In this method, the VIDAS device is used.^17^ In the present study, we used the ELISA method to measure the serum levels of T_3_ (kit sensitivity, 0.2 ng/mL; intra assay coefficients of variation [CV], 7.8%; and inter assay CV, 9.2%), T_4_ (kit sensitivity, 1 μg/dL; intra assay CV, 5.8%; and inter assay CV, 7.7%), TSH (kit sensitivity, 0.15 μIU/mL; intra assay CV, <15%; and inter assay CV, <15%), FSH (kit sensitivity, 2.5 mIU/mL; intra assay CV, <15%; and inter assay CV, <15%), LH (kit sensitivity, 0.5 mIU/mL; intra assay CV, <15; and inter assay CV, <15) and testosterone (kit sensitivity, 0.06 ng/mL; intra assay CV, 6.7%; and inter assay CV, 8.9%).

### 2.8 Histological studies

Immediately after blood sampling, by making a longitudinal incision in the scrotum, the testicles were removed with tweezers and its extra tissues were removed, and the tissue sections were carefully prepared and were studied using an optical microscope, first with a 10 and then with a 40 eyepiece. In this study, direct observation by light microscope and comparison of cell density in experimental groups with the control group was done. For morphometric analysis, 10 fields of view from each slide were randomly selected under the microscope and photographed by a digital camera. The photos were transferred to the computer and the diameter of the seminiferous tubules was measured using the Image Tool computer software, and the cells were counted visually under a light microscope with a magnification of 400. To count these cells, after preparing slides, the samples were observed by a microscope equipped with a camera. 10 sections of seminiferous tubules were selected from each slide sample corresponding to each mouse in different places of the field of view and counted, then the average of each mouse was taken.

### 2.9 Statistical analysis

The normality of the obtained data was evaluated using the Shapiro–Wilk test. Then the average data were presented as mean ± standard deviation (SD). Using the mean data, one‐way ANOVA and Tukey's post hoc test were performed to compare two by two between groups regarding each group and the differences between them were investigated. The criterion of significant difference between the data was considered as *p* < .05.

## 3 RESULTS

### 3.1 Histopathological examination of testis from the different experimental groups

**FIGURE 1:**
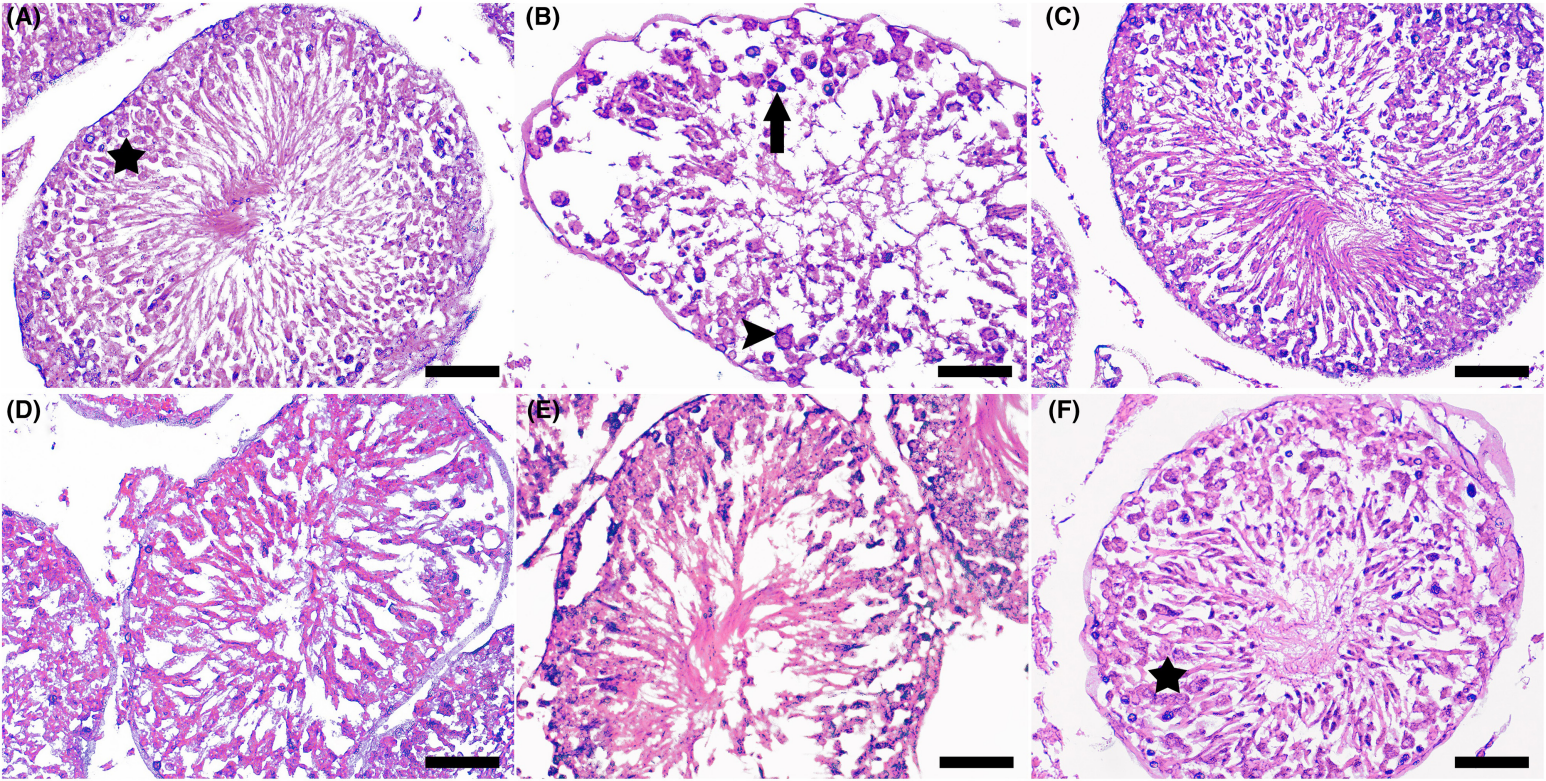
Histopathological examination of testis from the different experimental groups. Section from control group shown normal microscopic structure of spermatogenic tubules and spermatogonial cells (asterisk) (A); the cross‐section of 6‐n‐propylthiouracil (PTU) group showed that spermatogenic tube has an abnormal shape and atrophy and severe reduction of cell population as well as separation of spermatogonia (arrow) and Sertoli (arrowhead) cells are observed (B); the cross‐section from levothyroxine (LEVO) group has shown the normal microscopic state of the tissue (C); the cross‐section from *Elettaria cardamomum* extract (ECE) 100 group, treatment with this dose of ECE had a negligible effect on the microscopic structure of the spermatogenic tube tissue (D); the cross‐section from ECE 200 group, treatment with this dose of ECE had little effect on the microscopic structure of the spermatogenic tube tissue (E), the cross‐section from ECE 400 group, treatment with this dose of ECE has a significant effect on the improvement of the microscopic structure of the spermatogenic tube tissue, so that the damaged tube lining tissue (star) is regenerating (F). Haematoxylin and eosin ×400 magnification, scale bar = 50 μm.

The cross‐section of spermatogenic tubules have shown in the Figure 1A–F. As shown in the Figure 1A control has normal microscopic structure of spermatogenic tubules and spermatogonial cells. The cross‐section of PTU group showed that spermatogenic tube has an abnormal shape and atrophy and severe reduction of cell population as well as separation of spermatogonia and Sertoli cells are observed (Figure 1B). In the group receiving levothyroxine the normal microscopic state of the tissue has shown (Figure 1C). the cross‐section from ECE 100 group has shown in the Figure 1D, treatment with this dose of ECE had a negligible effect on the microscopic structure of the spermatogenic tube tissue. The cross‐section from ECE 200 group has shown in the Figure 1E, treatment with this dose of ECE had little effect on the microscopic structure of the spermatogenic tube tissue. The cross‐section from ECE 400 group has shown in the Figure 1F, treatment with this dose of ECE has a significant effect on the improvement of the microscopic structure of the spermatogenic tube tissue, so that the damaged tube lining tissue is regenerating.

### 3.2 Effect of *E. cardamomum* extract on the thyroid hormones and thyroid‐stimulating hormone

The test of between‐subject effects for T_3_, T_4_, and TSH were (*F* (5,36) = 12.50 and *p*‐value < .001; *F* (5,36) = 56.89 and *p*‐value < .001; *F* (5,36) = 60.05 and *p*‐value < .001, respectively), the main variable effect of ECE on T_3_, T_4_, and TSH level in the blood of male mice with 99% confidence is significant.

According to the results listed in Table 1, in the examination of the data obtained from the T_3_, T_4_, and TSH, the comparison between the control group and the PTU group shows a significant difference between them, and treatment with PTU significantly decreased the serum level of T_3_ and T_4_ (*p* < .05), and significantly increased the serum level of TSH (*p* < .05). Treatment with ECE reverse the effect of PTU and has led to a significant increase in the serum level of T_3_ and T_4_ (*p* < .05), and significant decrease in the serum level of TSH (*p* < .05) compared to the PTU group.

**TABLE 1:** Serum concentration of T_3_, T_4_ and thyroid‐stimulating hormone (TSH) in the different experimental groups.

Description	Groups					
	Control	PTU	LEVO	ECE 100	ECE 200	ECE 400
T_3_ (nmol/L)	2.94 ± 0.92	0.67 ± 0.38^a^	1.65 ± 0.58^a^	1.43 ± 0.64^a^	1.96 ± 0.49^b^	2.64 ± 0.57^b^
T_4_ (nmol/L)	89.28 ± 12.86	40.83 ± 3.98^a^	64.19 ± 5.74^a^ ^,^ ^b^	44.71 ± 5.02^a^ ^,^ ^c^	49.28 ± 4.71^a^ ^,^ ^c^	86.22 ± 8.35^b^ ^,^ ^c^
TSH (mU/mL)	3.30 ± 0.88	9.82 ± 1.21^a^	3.26 ± 1.09^b^	7.59 ± 1.00^a^ ^,^ ^b^ ^,^ ^c^	3.66 ± 0.70^b^	2.63 ± 1.07^b^

*Note*: Values represent as mean ± standard deviation of seven mice in each group.

Abbreviations: ECE, *Elettaria cardamomum* extract; LEVO, levothyroxine; PTU, 6‐n‐propylthiouracil.

^a^ Statistically different from control (*p* < .05).

^b^ Statistically different from PTU (*p* < .05).

^c^ Statistically different from LEVO (*p* < .05).

### 3.3 Effect of *E. cardamomum* extract on the sexual hormones

The test of between‐subject effects for testosterone, FSH, and LH were (*F* (5,36) = 40.67 and *p*‐value < .001; *F* (5,36) = 153.70 and *p*‐value < .001; *F* (5,36) = 216.40 and *p*‐value < .001, respectively), the main variable effect of ECE on testosterone, FSH, and LH level in the blood of male mice with 99% confidence is significant.

As shown in the Table 2, in the examination of the data obtained from the testosterone, FSH, and LH, the comparison between the control group and the PTU group shows a significant difference between them, and treatment with PTU significantly decreased the serum level of testosterone (*p* < .05), and significantly increased the serum level of FSH and LH (*p* < .05). Treatment with ECE reverse the effect of PTU and has led to a significant increase in the serum level of testosterone (*p* < .05), and significant decrease in the serum level of FSH and LH (*p* < .05) compared to the PTU group.

**TABLE 2:** Serum concentration of testosterone, follicle‐stimulating hormone (FSH) and luteinizing hormone (LH) in the different experimental groups.

Description	Groups					
	Control	PTU	LEVO	ECE 100	ECE 200	ECE 400
Testosterone (ng/mL)	2.58 ± 0.59	0.81 ± 0.17^a^	0.82 ± 0.09^a^	0.84 ± 0.20^a^	1.33 ± 0.44^a^	2.50 ± 0.34^b^ ^,^ ^c^
FSH (IU/L)	237.30 ± 19.82	467.90 ± 22.34^a^	444.70 ± 23.58^a^	447.00 ± 26.41^a^	349.60 ± 24.89^a^ ^,^ ^b^ ^,^ ^c^	248.70 ± 13.06^b^ ^,^ ^c^
LH (IU/L)	3.41 ± 0.39	7.79 ± 0.42^a^	8.47 ± 0.36^a^ ^,^ ^b^	7.78 ± 0.57^a^ ^,^ ^c^	5.69 ± 0.31^a^ ^,^ ^b^ ^,^ ^c^	3.74 ± 0.25^b^ ^,^ ^c^

*Note*: Values represent as mean ± standard deviation of seven mice in each group.

Abbreviations: ECE, *Elettaria cardamomum* extract; LEVO, levothyroxine; PTU, 6‐n‐propylthiouracil.

^a^ Statistically different from control (*p* < .05).

^b^ Statistically different from PTU (*p* < .05).

^c^ Statistically different from LEVO (*p* < .05).

### 3.4 Effect of *E. cardamomum* extract on the sperm count and gonadosomatic index

The test of between‐subject effects for sperm counts and GSI were (*F* (5,36) = 25.90 and *p*‐value < .001; *F* (5,36) = 24.81 and *p*‐value < .001, respectively), the main variable effect of ECE on sperm counts and GSI in male mice with 99% confidence is significant.

**FIGURE 2:**
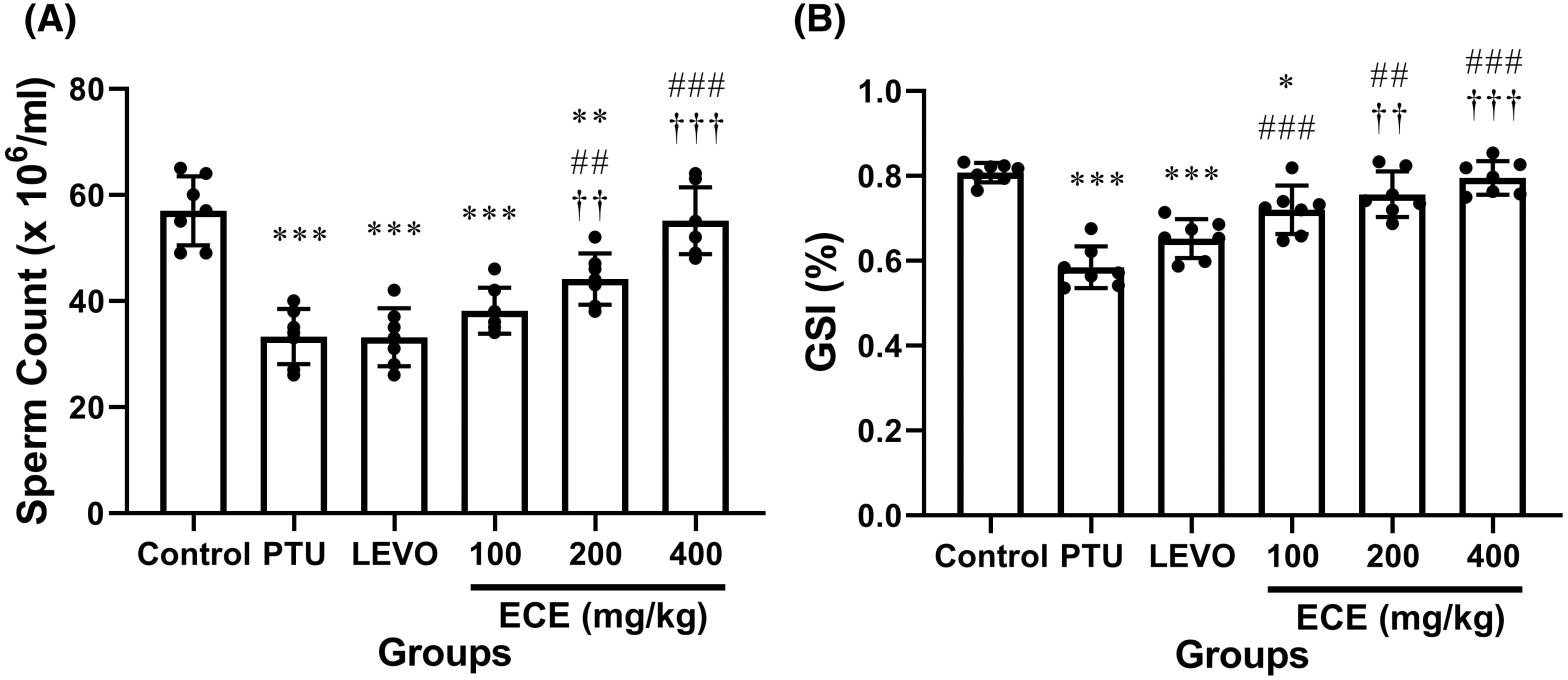
Comparison of the sperm count (A), and gonadosomatic index (GSI) (B) in the different experimental groups. Data represent as mean ± standard deviation of seven mice in each group. *p < .05, **p < .01, ***p < .001 vs. control; ^##^ *p* < .01, ^###^ *p* < .001 vs. 6‐n‐propylthiouracil (PTU); ^††^ *p* < .01, ^†††^ *p* < .001 vs. levothyroxine (LEVO). ECE, *Elettaria cardamomum* extract.

Figure 2A,B show the sperm counts and GSI factors, in the examination of the data obtained from the sperm counts and GSI, the comparison between the control group and the PTU group shows a significant difference between them, and treatment with PTU significantly decreased the sperm counts and GSI (*p* < .05). Treatment with ECE reverse the effect of PTU and has led to a significant increase in the sperm counts and GSI (*p* < .05) compared to the PTU group.

## 4 DISCUSSION

This study was conducted in order to determine the effect of ECE on the process of spermatogenesis in hypothyroid male mice induced by PTU. The results of this study show that ECE has led to a change in the function of the testis and the hormonal axis of the hypophysis of the gonad in the group treated with ECE. In the present study, the group receiving ECE had a significant increase in GSI. Since body weight and testicles are influenced by testosterone hormone, according to the significant increase in testosterone hormone in this study and the subsequent increase in the number of Leydig cells and spermatogonial cells, spermatocytes and spermatids. Previous study has shown that testosterone has a direct effect on Sertoli cells and the secretion of tubular fluid and several proteins such as growth factor and transferrin play a special role in the nutrition of dividing sex cells, sexual organs and finally sperm production.^18^ In this way, considering the important role of testosterone hormone in the process of spermatogenesis, it is clear that if this hormone increases, the number of sperms increases subsequently. The results of the present study also confirm a significant increase in the number of sperms after treatment with ECE.

Previous studies confirm the role of thyroid hormones on infertility disorders in men.^19^ Among these studies, it is possible to mention the research of Romano and his colleagues on the changes of testicular tissue and hormones in rats with congenital hypothyroidism in early life.^20^ The results show the reducing effects of hypothyroidism on the production of steroids and spermatogenesis and as a result, reducing fertility in these animals. These findings can be due to the reduction of pituitary stimulation and testicular response. In the research conducted in 2008 by Krassas et al. on the effect of hypothyroidism on the phenomenon of spermatogenesis in humans, it was shown that sperm morphology is significantly affected by hypothyroidism. Of course, sperm movement may also be affected. Therefore, hypothyroidism has a negative effect on the process of spermatogenesis.^21^

Research shows that phytoestrogens in plant extracts have the ability to bind to oestrogen receptors^22^ and by creating a negative feedback on LH, it reduces the amount of testosterone.^23^, ^24^ Phytochemical studies show that cardamom seeds have chemical compounds such as alpha‐terpineone, diterpenes, menthone, limonene, sabinene, heptane, myrcene, beta‐nerolidol, linalool, beta‐pinene, alpha pinene, eugenyl acetate, gamma‐sitosterol, citronellol, terpinene and pinene. Chemical analysis shows that cardamom contains alkaloid, flavonoid, saponin, sterol and tannin. The citronellol composition of this plant is known as a sedative and anti‐depressant.^25^

In the study of Taheri and Mirazi^14^ on the effect of green cardamom extract on the serum level of thyroid hormones, there was a significant increase in the plasma level of thyroid hormones T_3_ & T_4_ and a significant decrease in the plasma level of TSH hormone in male mice due to the addition of plant seed extract. With this result, it can be acknowledged that the hydroalcoholic extract of cardamom plant can effectively stimulate the thyroid gland and increase its secreted hormones. Since cardamom seeds have special compounds such as diterpenes with antioxidant properties. Therefore, the cardamom seed extract has anti‐peroxidase and antioxidant properties, it is possible that through its anti‐peroxidase properties, it was able to increase T_3_ and T_4_ hormones.^14^ According to these findings, it is possible that the effects of green cardamom plant on the process of spermatogenesis in the present study are due to the improvement of thyroid gland function. Therefore, the results of the present research are consistent with the results of Taheri and Mirazi's research and are in the same direction. In the research conducted by Rezaei et al. (2016) on the effect of the hydroethanolic extract of the green cardamom fruit on the serum level of gonadotropins and testosterone in adult male rats induced with lead acetate, it was shown that the hydroalcoholic extract of the green cardamom fruit is probably by the elimination mechanism and the neutralization of free radicals caused a decrease in the destructive effects of lead on the testicles and an increase in the secretion of testosterone, followed by the return of the serum levels of LH and FSH hormones to normal. Considering the side effects of drugs, the extract of this plant can be considered as a natural and safer antioxidant.^26^

Histological examinations and counting of spermatocytes inside the seminiferous tubules showed that in the experimental group these cells had a significant increase compared to the PTU group. According to the conducted study, the process of spermatogenesis and the transition from germ cells to reaching the maturity stage of sex cells depends on being protected from pathological and cytotoxic lesions that threaten this phenomenon.^27^ It can be concluded that one of the possible mechanisms of the effect of the ECE on the testicular tissue is the increase in the number of Leydig cells and as a result the increase of male sex hormones and the increase of blood supply to the testicular tissue through veins. Both of these cases have caused an increase in sperm cells through the effect on the Sertoli cell, which controls the process of spermatogenesis. Our study has some limitations that should be considered. As limitations, our study did not evaluate the other biochemical and molecular parameters which were very important such as antioxidant/oxidant statue or inflammatory cytokines, and gene expression of steroidogenic pathway due to limited funding, and sample size. Therefore, supplementary studies need to justify the result and beneficial effect of ECE on the hypothyroidism condition.

## 5 CONCLUSION

According to the results of the present research, the ECE decreased the hypothyroid inhibitory effects on testicular tissue, increased the number of sex cells, and increased testosterone secretion. Considering the side effects of drugs, the extract of this plant can be considered as a natural and safer antioxidant.

## AUTHOR CONTRIBUTIONS

**Bahar Atabaki:** Conceptualization (equal); data curation (equal); formal analysis (equal); funding acquisition (equal); investigation (equal); methodology (equal); project administration (equal); resources (equal); software (equal); supervision (equal); validation (equal); visualization (equal); writing – original draft (equal); writing – review and editing (equal). **Naser Mirazi:** Conceptualization (equal); data curation (equal); formal analysis (equal); funding acquisition (equal); investigation (equal); methodology (equal); project administration (equal); resources (equal); software (equal); supervision (equal); validation (equal); visualization (equal); writing – original draft (equal); writing – review and editing (equal). **Abdolkarim Hosseini:** Conceptualization (equal); data curation (equal); formal analysis (equal); funding acquisition (equal); investigation (equal); methodology (equal); project administration (equal); resources (equal); software (equal); supervision (equal); validation (equal); visualization (equal); writing – original draft (equal); writing – review and editing (equal). **Abdolrahman Sarihi:** Conceptualization (equal); data curation (equal); formal analysis (equal); funding acquisition (equal); investigation (equal); methodology (equal); project administration (equal); resources (equal); software (equal); supervision (equal); validation (equal); visualization (equal); writing – original draft (equal); writing – review and editing (equal). **Zahra Izadi:** Conceptualization (equal); data curation (equal); formal analysis (equal); funding acquisition (equal); investigation (equal); methodology (equal); project administration (equal); resources (equal); software (equal); supervision (equal); validation (equal); visualization (equal); writing – original draft (equal); writing – review and editing (equal). **Alireza Nourian:** Conceptualization (equal); data curation (equal); formal analysis (equal); funding acquisition (equal); investigation (equal); methodology (equal); project administration (equal); resources (equal); software (equal); supervision (equal); validation (equal); visualization (equal); writing – original draft (equal); writing – review and editing (equal).

## FUNDING INFORMATION

This study did not receive any external funding.

## CONFLICT OF INTEREST STATEMENT

The authors declare no conflict of interest.

## Data Availability

The data that support the findings of this study are available from the corresponding author upon reasonable request.
